# Changes in complex spike activity during classical conditioning

**DOI:** 10.3389/fncir.2014.00090

**Published:** 2014-08-05

**Authors:** Anders Rasmussen, Dan-Anders Jirenhed, Daniel Z. Wetmore, Germund Hesslow

**Affiliations:** ^1^Department of Experimental Medical Science, Associative Learning Group, Lund UniversityLund, Sweden; ^2^Linneaus Center CCL, Lund UniversityLund, Sweden; ^3^Department of Physics, James H. Clark Center for Biomedical Engineering and Sciences, Stanford UniversityStanford, CA, USA

**Keywords:** inferior olive, nucleo-olivary pathway, complex spikes, purkinje cells, *in vivo* electrophysiology, eyeblink conditioning, oscillations, interstimulus interval

## Abstract

The cerebellar cortex is necessary for adaptively timed conditioned responses (CRs) in eyeblink conditioning. During conditioning, Purkinje cells acquire pause responses or “Purkinje cell CRs” to the conditioned stimuli (CS), resulting in disinhibition of the cerebellar nuclei (CN), allowing them to activate motor nuclei that control eyeblinks. This disinhibition also causes inhibition of the inferior olive (IO), via the nucleo-olivary pathway (N-O). Activation of the IO, which relays the unconditional stimulus (US) to the cortex, elicits characteristic complex spikes in Purkinje cells. Although Purkinje cell activity, as well as stimulation of the CN, is known to influence IO activity, much remains to be learned about the way that learned changes in simple spike firing affects the IO. In the present study, we analyzed changes in simple and complex spike firing, in extracellular Purkinje cell records, from the C3 zone, in decerebrate ferrets undergoing training in a conditioning paradigm. In agreement with the N-O feedback hypothesis, acquisition resulted in a gradual decrease in complex spike activity during the conditioned stimulus, with a delay that is consistent with the long N-O latency. Also supporting the feedback hypothesis, training with a short interstimulus interval (ISI), which does not lead to acquisition of a Purkinje cell CR, did not cause a suppression of complex spike activity. In contrast, observations that extinction did not lead to a recovery in complex spike activity and the irregular patterns of simple and complex spike activity after the conditioned stimulus are less conclusive.

## Introduction

**Figure 1:**
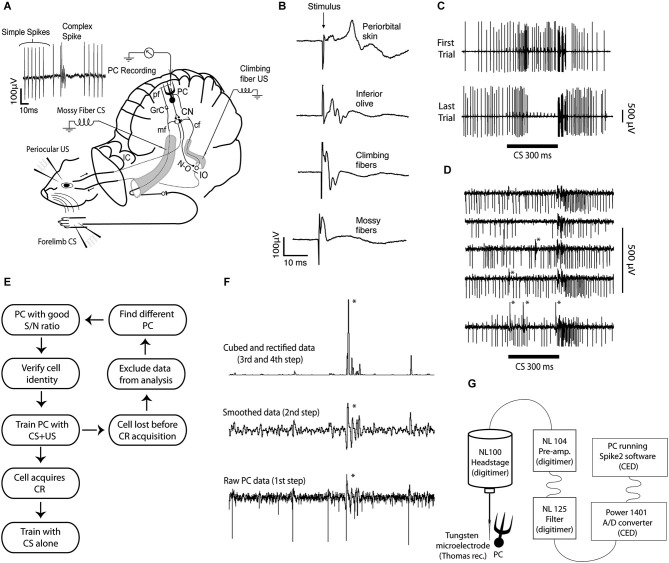
**Experimental setup, field and single unit recordings, protocol diagram, method for sorting complex spikes, and recording setup. (A)** Extracellular Purkinje cell recordings (PC Recording) were made from lobule HVI, in the left cerebellar cortex. The conditional stimulus (CS), consisted of forelimb or mossy fiber stimulation. The unconditional stimulus (US), consisted of periocular or climbing fiber stimulation. The signal generated by the forelimb CS enters the cerebellum via mossy fibers (mf), in the inferior (not shown) and the middle cerebellar peduncle. They synapse on granule cells (GrC), whose axons, the parallel fibers (pf), synapse on Purkinje cells (PCs), in the cerebellar cortex. The signal generated by the periocular US is transmitted to the inferior olive (IO), which sends climbing fibers (cf) through the inferior cerebellar peduncle, to Purkinje cells. Purkinje cells project to the cerebellar nuclei (CN), which in turn project to motor nuclei that control eye muscles, as well as to the IO, via the GABAergic nucleo-olivary pathway (N-O). **(B)** Field potentials recorded in the C3 zone, on the surface of the cerebellar cortex, following stimulation of different sites along the CS and US pathways. **(C)** The response of a single Purkinje cell before and after acquisition of a conditioned pause response or Purkinje cell CR. **(D)** Records from a Purkinje cell illustrating an increased complex spike probability in the early part of the CS. **(E)** Schematic diagram illustrating the strategy for obtaining data. **(F)** Illustration of the semiautomatic procedure used to sort complex spikes. Because a complex spike typically has one or more secondary spikes, smoothing the record will make them stand out. If the record is subsequently rectified, and then squared or cubed, these differences will be amplified. Still visual inspection is usually necessary to ensure accuracy. **(G)** Data acquisition setup.

**Figure 2:**
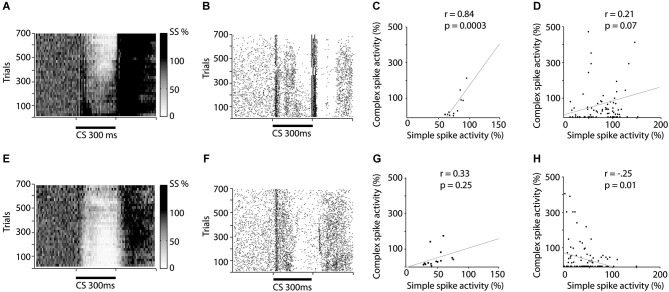
**Raster and scatter plots illustrating training induced changes in simple and complex spike activity.** **(A–D)** illustrates changes in activity during acquisition while **(E–H)** illustrates changes during extinction. (**A** and **E**) Relative change in simple spike firing during training. The darkness of each square represent the average simple spike frequency in that time period, divided by the simple spike frequency 600 ms before CS onset. Each square represents 10 trials and 10 ms. (**B** and **F**) Raster plot depicting complex spike activity during training. Each dot represents a complex spike and each row represents one trial. (**C** and **G**) Correlation between the average simple spike frequency and complex spike frequency. Each dot represents the relative simple spike frequency 150–250 ms after CS onset, in a 50 trial bin and the relative complex spike activity 200–300 ms after CS onset in the same 50 trials. (**D** and **H**) Correlation between un-pooled simple and complex spike firing.

In eyeblink conditioning, repeated pairings of an originally neutral conditional stimulus (CS) and an unconditional, reflex-eliciting stimulus (US), results in acquisition of a conditioned response (CR), occurring just before the expected US onset. This CR can be extinguished by repeatedly presenting the CS alone (Kehoe and Macrae, 2002). It is well established that eyeblink conditioning is critically dependent on the cerebellum (McCormick and Thompson, 1984; Bracha et al., 2009), and that the cerebellar cortex is necessary for generating adaptively timed CRs (Yeo et al., 1984; Hesslow and Yeo, 2002; Kellett et al., 2010). During conditioning, Purkinje cells, which provide the sole output from the cerebellar cortex, acquire a pause response, a “Purkinje cell CR” (Figures 1C, 2A,E), that shares many features with learned eyeblinks (Jirenhed et al., 2007; Jirenhed and Hesslow, 2011a,b). Purkinje cells receive information about the CS via mossy fibers, synapsing on granule cells that send parallel fibers to Purkinje cell dendrites (Steinmetz et al., 1986; Hesslow et al., 1999). Information about the US arrives via the climbing fibers originating in the inferior olive (IO; Mauk et al., 1986). Since Purkinje cells are inhibitory, a pause response will cause disinhibition of their target neurons in the cerebellar nuclei (CN; De Zeeuw and Berrebi, 1995), allowing them to initiate a motor response (Hesslow, 1994a; Heiney et al., 2014).

In addition, disinhibition of the CN causes activation of the nucleo-olivary pathway (N-O; Graybiel et al., 1973). There is compelling evidence that this pathway is GABAergic (de Zeeuw et al., 1988; Nelson and Mugnaini, 1989; Barmack, 1996) and that it exerts a strong inhibitory effect on the IO (Hesslow, 1986; Andersson et al., 1988; Svensson et al., 2006; Bazzigaluppi et al., 2012; Bengtsson and Hesslow, 2013). The evidence also suggests that the N-O fibers form a closed loop, that is, they target those olivary cells that are part of the same microcomplex (Apps and Garwicz, 2005). It was proposed by Andersson et al. (1988) that the N-O fibers allow the cerebellum to give negative feedback to the IO, either for stabilizing the background firing of Purkinje cells or for stabilizing learning (Andersson and Hesslow, 1987; Andersson et al., 1988). In accordance with the view that the climbing fibers from the IO transmit teaching signals to the cerebellum, such as the US in classical conditioning, an output signal from the CN to the motor system would be accompanied by an inhibitory signal to the IO to suppress the teaching command and further learning.

A large number of studies have pursued this idea and provided evidence for the feedback hypothesis. Thus, it has been shown that the IO is indeed suppressed when conditioned responses (CRs) are being emitted and that activation of the N-O pathway can cause extinction of a learned response (Sears and Steinmetz, 1991; Hesslow and Ivarsson, 1996; Apps and Lee, 2002; Bengtsson et al., 2007; Chaumont et al., 2013). Other behavioral phenomena, related to learning, such as extinction (Medina et al., 2002) and blocking (Kim et al., 1998), have also been ascribed to N-O inhibition.

Although a lot has been learned about the N-O projection (Bengtsson and Hesslow, 2013), many important questions have remained unanswered: How does the learned Purkinje cell CR compare to N-O stimulation in its ability to influence IO activity? Is the delay between the Purkinje cell CR and the IO inhibition consistent with the long delay in the N-O pathway (Hesslow, 1986; Svensson et al., 2006)? Does IO activity increase during extinction, when the Purkinje cell CR disappears? Does reduced simple spike firing in a Purkinje cell cause inhibition of IO neurons projecting to that same Purkinje cell? Studying changes in complex spike frequency during acquisition and extinction of a Purkinje cell CR can address these questions because complex spikes reflect IO activity. Further clarification of the relationship between learned changes in Purkinje cell activity and complex spike frequency can be obtained by analyzing changes after training with short interstimulus intervals (ISIs), which on a behavioral level does not result in learning (Salafia et al., 1980). Mirroring the behavior, Purkinje cell CRs are not acquired for ISIs below ~100 ms, and using a 50 ms ISI even causes increased Purkinje cell activity (Wetmore et al., 2014). Is this increase in simple spike frequency associated with a similar increase in complex spike frequency? This study aims to answer these questions by examining the effect of learned changes in Purkinje cell simple spikes on complex spike activity.

Information about olivary activity during conditioning is also important for evaluating the main alternatives to the feedback hypothesis. It has been suggested that the N-O pathway controls coupling between olivary cells and that firing in specific ensembles of these cells are the main drivers of well timed movements (Jacobson et al., 2008). The temporal pattern of complex spikes during one of the best-studied examples of an adaptively timed cerebellar response would provide a test of this hypothesis.

## Materials and methods

The experimental setup is illustrated in Figure 1A. Subjects were 28 male ferrets (0.6–1.5 kg), initially anesthetized in a mixture of O_2_ and air, with 1.5–2% isoflurane (Baxter Medical, Kista, Sweden), subsequently replaced by propofol (10 mg/ml Diprivan; AstraZeneca, Södertälje, Sweden) intravenously. Blood pressure, CO_2_, and temperature were kept within physiological limits throughout the experiment. After the head was fixed in a stereotaxic frame and the skull opened on the left side, the caudal two thirds of the cerebral hemisphere was removed by aspiration, exposing the anterior cerebellar cortex and the colliculi. Animals were decerebrated by sectioning the brainstem with a blunt spatula 1–2 mm rostral to the superior colliculus. After decerebration, anesthesia was discontinued. With the cerebellum and colliculi exposed, a pool was constructed of cotton reinforced agar and filled with high-density perfluoro carbon liquid (FC-40 Fluorinert; 3M, Zwijndrecht, Belgium). To achieve high stability, ferrets were curarized, artificially ventilated, and were kept hanging by the spine, with the head fixed in the stereotaxic frame. A bilateral pneumothorax was performed in order to minimize chest movements. After removal of the dura, the cerebellar surface was covered with agarose gel (20 mg/ml) to provide recording stability and prevent edema near the site of recording. For additional details on surgical procedures see Jirenhed et al. (2007). This study has been reviewed and approved by the local Swedish Ethical Committee.

### Stimulation of cerebellar afferents

While tracking for all types of cerebellar afferents (IO, inferior and middle cerebellar peduncle), single stimulus pulses were applied and field potentials were recorded in a blink controlling area of the C3 zone of hemispheral lobule VI, identified by previously established criteria (Hesslow, 1994a,b). Only when stimulation elicited a field potential with the right latency and shape (Figure 1B), and at a relatively low stimulus intensity did we proceed with Purkinje cell recordings. Direct stimulation of cerebellar afferents was done with tungsten electrodes (diameter, 100 μm; deinsulated tip, 50 μm). The effectiveness of all stimulation sites and stimulus intensities were verified again and adjusted if necessary when recording the activity of single Purkinje cells.

The CS consisted of either peripheral forelimb stimulation or direct mossy fiber stimulation. The forelimb CS was a 300 ms, 50 Hz stimulus train applied to the ipsilateral forelimb skin (1.5–2.0 mA, 0.5 ms pulse duration). The mossy fiber CS also consisted of a 300 ms, 50 Hz stimulus train (90–200 μA, 0.1 ms pulse duration). The US consisted of climbing fiber stimulation (15–90 μA, 0.1 ms pulse duration), direct IO stimulation (70–320 μA, 0.1 ms pulse duration), or periorbital stimulation (3 mA, 0.5 ms pulse duration). Central US stimulation (climbing fibers and IO) consisted of two 10 ms stimulus trains delivered with a 20 ms interval, each consisting of five pulses at 500 Hz. This stimulation protocol was used because it mirrors the natural firing pattern of the IO, which consists of high frequency bursts (Armstrong and Rawson, 1979; Maruta et al., 2007; Mathy et al., 2009). Peripheral stimuli can sometimes elicit a couple of consecutive bursts with an interval of about 20 ms between each burst (Ekerot et al., 1987). Moreover, these US parameters reliably results in the acquisition of Purkinje cell CRs (Jirenhed et al., 2007), which is not the case if the US consists of a single pulse (Rasmussen et al., 2013). In the remainder of this manuscript we will not distinguish between different types of CS and US stimulation because these have all been shown to induce Purkinje cell CRs.

### Training protocols

Because this article is based on data from several different projects, the protocols used across experiments differ slightly from one another. The protocol for the 12 ferrets conditioned with a 300 ms ISI was analogous to the protocols used in previous experiments in our laboratory (Hesslow and Ivarsson, 1994; Hesslow et al., 1999; Jirenhed et al., 2007), where the last pulse in the CS train coincides with the first pulse in the US train. The intertrial interval was 15 s. When a cell meeting our pre-defined criteria (see below) had been found, paired CS-US presentations were applied until a Purkinje cell CR developed (Figure 1E). If the cell was lost during training, we discarded data from that cell and tried to find a different cell in which we could study extinction. Following acquisition of a clear Purkinje cell CR we switched to CS alone presentations in order to follow the cell during extinction. The CS and US parameters were kept constant for the 16 ferrets trained with shorter ISIs. Before any paired CS-US presentations were given, 40–60 CS alone trials were recorded. This allowed us to estimate Purkinje cell activity in naïve cells. After the initial CS alone session, paired CS-US presentations, with a 50 or 150 ms ISI, were delivered for approximately 1 h or 240 trials. After this, another CS alone session followed, and then we proceeded with another paired session. This approach, with interspersed CS alone sessions, allowed us to estimate changes in Purkinje cell activity without influence from the US.

### Purkinje cell recordings

Extracellular single-unit recordings of Purkinje cells, identified by the presence of complex spikes, were performed using quartz glass-coated platinum–tungsten microelectrodes with pulled and ground tips; 30–40 μm metal core diameter (Thomas Recording, Giessen, Germany). All Purkinje cells were located in a blink-controlling area of the C3 zone of the ipsilateral hemispheral lobule VI (Hesslow, 1994a,b). Mossy fiber input from the CS could be verified by increases in simple spike activity on presentation of a 300 ms stimulus train. The following recording strategy was used to obtain and follow single-cell records: (1) find Purkinje cell (identified by presence of spontaneous complex spikes) in a naive animal; (2) verify identity as eyeblink-controlling cell in C3 by monitoring responses to periorbital stimulation; (3) verify afferent mossy and climbing fiber inputs from the prospective CS and US; (4) establish effective stimulation thresholds for CS and US; (5) start paired CS–US presentations and record during acquisition phase. (a) If recording is sufficiently long and stable to observe a clear learning effect, proceed to step 6. (b) If cell is lost during training, continue with paired CS–US presentations, locate another cell, and go back to step 1; and (6) if an identified Purkinje cell is found that reliably exhibits a clear change in simple spike responses to the CS, proceed with extinction (CS-alone trials), or switch to a different ISI. A record was judged to be from the same Purkinje cell only if (1) the cell was observed continuously throughout the recording; (2) there were no spikes of different but comparable amplitude present at any time; and (3) there were no sudden changes in spike amplitude. The position of the recording electrode occasionally had to be adjusted, but this could usually be done without violating these criteria.

### Data acquisition

The signal from the microelectrode was fed into a pre-amplifier (NL104) + filter module (NL125), via a NL100 headstage, all from Digitimer Ltd. To eliminate remaining 50 Hz noise, the signal was passed through a humbug (Digitimer Ltd), before entering a Power 1401 analog/digital converter interface (Cambridge Electronics Design), which sampled the signal at 30 kHz and passed it on, via a USB interface, to a PC running Spike2 v7 software (Figure 1G). Online analysis and offline spike sorting was performed using Spike2, version 7 (Cambridge Electronics Design), and subsequent data analysis was done using custom made Matlab scripts (MathWorks).

### Complex spike identification and data analysis

Separating complex spikes from simple spikes and stimulus artifacts in Purkinje cell records involves a number of difficulties: (1) complex spikes and simple spikes may have similar waveforms; (2) the waveform of complex spikes differs from cell to cell; (3) complex spike waveforms can change over time within a single record; and (4) the secondary spikes in the complex spikes can vary substantially, especially if the complex spike occurs in proximity to a simple spike or a stimulus artifact. Although sorting complex spikes is difficult, our lab has several decades of experience with studying the IO and examining complex spikes. To minimize the risk of misses and false positives we only used records with a good signal to noise ratio and with distinct complex spikes. The following strategy, illustrated in Figure 1F, was used to make sorting easier: (1) remove DC fluctuations to get a flat baseline; (2) apply a smoothing function that replaces all data-points with the mean or the median of a specified time window around that data-point (smoothing amplifies complex spikes because, compared to simple spikes, the complex spike waveform deviates from the baseline for a longer period of time); (3) square or cube waveform to further amplify the amplitude difference between complex spikes, simple spikes and stimulus artifacts; and (4) set appropriate thresholds to sort out the complex spikes. It should be stressed that, to ensure accurate complex spike identification, all complex spikes were verified by visual inspection of the recordings. To guard against experimenter bias we sometimes had two individuals sort the same data.

Analyzing changes in complex spike firing rate during conditioning has its own difficulties. Compared to simple spikes, complex spikes occur at a very low frequency, and therefore more data are required to find patterns. Moreover, complex spikes can vary from cell to cell and may also vary over time within a single record (this is true for simple spikes as well). To address these challenges, we split the Purkinje cell record into 50 trial bins. For each bin we normalized the complex spike activity during the CS by dividing it with the background activity, defined as the average activity 600 ms before the start of the CS. This allowed us to express the complex spike activity within different parts of the CS as a percentage of background activity. When analyzing changes in complex spike activity during acquisition and extinction we were mainly interested in changes during the last 100 ms of the CS. The reason was that the Purkinje cell CR peaks approximately 180 ms into the CS (Jirenhed et al., 2007), and any effects on complex spike frequency ought to occur after this. Moreover, we wanted to avoid interference from the CS facilitated complex spikes, which often occur early in the CS. For these two reasons the statistical analysis focused on the last 100 ms of the CS, although we also report observed changes in other time periods.

## Results

The analysis below is based on a total of 32 cells. Eight Purkinje cells, in eight animals, were recorded from the naïve state until a Purkinje cell CR had been acquired. Five of these eight were also recorded throughout the extinction phase. An additional four cells, which had not been followed during the entire acquisition phase, but which exhibited typical Purkinje cell CRs when found, were also included in the extinction dataset. The analysis of complex spike activity following training with a short ISI, was based on 20 cells trained with a 50, or 150 ms ISI.

### Complex spike activity during acquisition and extinction

The background frequency (mean ± sd) was 1.04 ± 0.53 Hz, for complex spikes and 69.9 ± 30.6 Hz for simple spikes. Repeated CS–US presentations result in acquisition of a Purkinje cell CR (Figure 2A), with the minimum simple spike activity occurring 180 ms after CS onset, when using a 300 ms ISI. If the CS is subsequently presented alone, this simple spike pause response is gradually extinguished (Figure 2E). Based on these results and previous observations suggesting that simple spike activity influences complex spike activity (Miall et al., 1998; Bengtsson and Hesslow, 2006; Rasmussen et al., 2008), we predicted that acquisition and extinction of a Purkinje cell CR would be associated with a corresponding change in complex spike activity. In other words, our prediction was that the acquisition phase would be associated with a gradually decreasing complex spike firing rate, towards the end of the CS–US interval, and that extinction would be associated with a corresponding increase in the complex spike firing rate.

**Figure 3:**
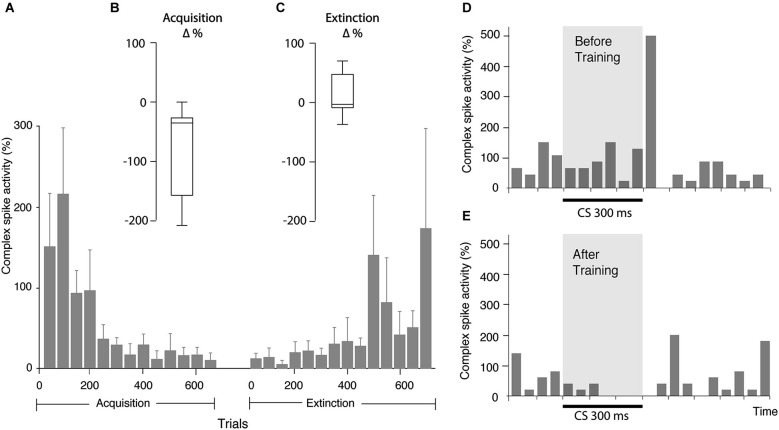
**Changes in complex spike activity as a result of training.** **(A)** Bar chart illustrating complex spike activity (mean ± SEM), relative to complex spike activity before CS onset, in consecutive bins, each consisting of 50 trials. Since all cells were not followed for the same number of trials, the bars representing the beginning of acquisition and extinction are averages of more cells than the bars representing the latter part of the process. Every bar is based on data from at least three cells. **(B)** Distribution of change in complex spike activity between the first 50 and last 50 trials of the acquisition phase (*n* = 8). **(C)** Distribution of the change in complex spike activity between the first 50 and last 50 trials of the extinction phase (*n* = 9). **(D)** Bar chart illustrating complex spike frequency in the first 100 trials in the acquisition phase. **(E)** Complex spike activity in the same cell in the first 100 trials in the extinction phase. Binsize is 50 ms.

In line with these predictions, seven of the eight cells undergoing acquisition showed decreased complex spike activity in the last 100 ms of the CS, following training. This decrease developed gradually as training progressed (Figures 2B, 3A,B). During the first 50 trials, the average complex spike frequency in the last 100 ms of the CS was 127%, relative to background frequency. During training, complex spike activity decreased steadily and after 400 trials it was down to 21%, relative to background. After 600 trials the average frequency was 14% relative to background. A Wilcoxon matched-pairs test confirmed that there was a difference in complex spike activity between the first and last 50 trials of acquisition (*W* = −28, *n*_1_ = *n*_2_ = 8, *p* = 0.0156, two-tailed).

Nine Purkinje cells, with distinct complex spikes, were recorded for a sufficient amount of time to observe extinction of the Purkinje cell CR (Figure 2E). Changes in complex spike activity during the extinction phase were characterized by a high degree of variability (Figures 2F, 3A,C). Only four of nine cells showed increased complex spike activity as a result of extinction. Given that these four cells were all among the five cells that we managed to hold for at least 650 trials, it remains a possibility that extinction of the complex spike suppression simply requires more time. Overall, there was no statistically significant change in complex spike activity during extinction (*W* = 17, *n*_1_ = *n*_2_ = 9, *p* = 0.3594, two-tailed).

If simple spike activity in Purkinje cells influence subsequent complex spike activity, one would expect a positive correlation between the simple and complex spike activity in the latter part of the CS. To test this we calculated the correlation between simple spike activity, 150–250 ms after CS onset, and complex spike activity, 200–300 ms after CS onset, in 50 trial bins throughout acquisition and extinction. The reason for using these time periods for simple and complex spikes was that the complex spike suppression tends to appear later than the simple spike pause. For acquisition there was indeed a positive correlation between the average number of simple spikes and the average number of complex spikes, in a given 50 trial bin (Figure 2C).

However, surprisingly, during the extinction phase there was no statistically significant correlation (Figure 2G). Because averaging over cells can mask effects, which may be present within cells, we also did an analysis on un-pooled data. This analysis revealed a marginally significant correlation during acquisition (Figure 2D), and a surprising negative correlation during the extinction phase (Figure 2H).

### CS facilitated complex spikes

In 10 of 12 Purkinje cells trained with 300 ms ISI, there was a substantial increase in complex spike activity in the first ~100 ms of the CS, which was particularly pronounced in the first 10–20 ms (Figures 1D, 2B,F). On average complex spikes appeared in this time window in 4 out of 10 trials, which is four times higher than the natural background firing-rate. The magnitude of this increase varied between cells. In a few cells there were no CS facilitated complex spikes, whereas other cells had CS facilitated complex spikes on almost every trial. This finding raised the question of whether these early CS facilitated complex spikes had any influence on the complex spike frequency in the latter part of the CS? Was the complex spike suppression in the last 100 ms of the CS simply a rebound inhibition, caused by early CS facilitated complex spikes?

Several observations suggested that this was not the case. The two cells that did not have CS facilitated complex spikes still exhibited a suppression of complex spikes in the last 100 ms of the CS (Figures 3D,E). Moreover, whereas the complex spike suppression developed gradually, the frequency of complex spikes in the early part of the CS did not change as training progressed. If the complex spike suppression was cause by the early increase in complex spikes, it should have been present when training began.

**Figure 4:**
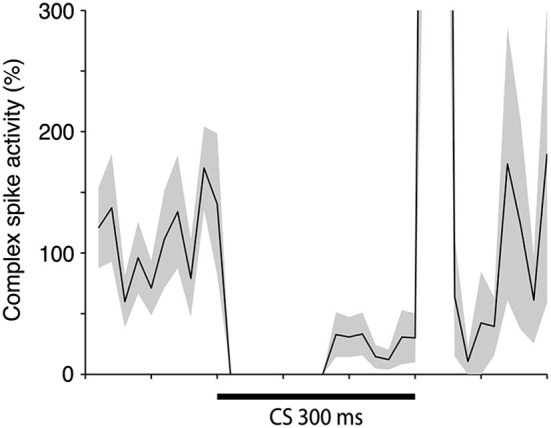
**Complex spike activity during the CS when all trials with CS facilitated complex spikes has been excluded.** Mean ± SEM of relative complex spike activity in 11 cells either at the end of acquisition (*n* = 7), or in the beginning of extinction (*n* = 4). One cell was excluded from this analysis because it displayed CS facilitated complex spikes on a high proportion of the trials.

In addition, we examined whether exclusion of trials with early complex spikes had any effect on the complex spike suppression in the last 100 ms of the CS. We found that even after excluding all trials with CS facilitated complex spikes, there was still a complex spike suppression in the end of the CS (Figure 4). Indeed, the average complex spike activity, after acquisition, in the last 100 ms of the CS was 16% in trials with one or more complex spikes in the early part of the CS and only 7% in trials without complex spikes in the early part of the CS. These observations show that the CS facilitated complex spikes did not cause the subsequent complex spike suppression.

The fact that the increase in complex spike activity, in the early part of the CS, was equally large throughout training also suggests that this increase cannot be the cause of the timed simple spike pause response that drives the overt CR. This point is further strengthened by the observation that whereas simple spike activity correlates with subsequent complex spike activity, there was no correlation between the complex spike activity and the subsequent simple spike activity (Table 1).

**Table 1:** Correlation between pooled and un-pooled simple and complex spike activity in different 100 ms time windows during the CS, in the acquisition phase (* *p* < 0.05, ** *p* < 0.01, *** *p* < 0.001).

**Simple spike time window**	**Complex spike time window**	**Delay Complex—Simple**	**Correlation, population**	**Correlation, cell−wise**
0–100 ms	200–300 ms	200 ms	*r* = 0.80, *r*_2_ = 0.63***	*r* = 0.25, *r*_2_ = 0.062*
50–150 ms	200–300 ms	150 ms	*r* = 0.85, *r*_2_ = 0.73***	*r* = 0.25, *r*_2_ = 0.064*
100–200 ms	200–300 ms	100 ms	*r* = 0.88, *r*_2_ = 0.78***	*r* = 0.25, *r*_2_ = 0.06*
150–250 ms	200–300 ms	50 ms	*r* = 0.84, *r*_2_ = 0.71***	*r* = 0.21, *r*_2_ = 0.045 ns
200–300 ms	200–300 ms	0 ms	*r* = 0.73, *r*_2_ = 0.54***	*r* = 0.13, *r*_2_ = 0.017 ns
0–100 ms	150–250 ms	150 ms	*r* = 0.56, *r*_2_ = 0.31*	*r* = 0.19, *r*_2_ = 0.036 ns
50–150 ms	150–250 ms	100 ms	*r* = 0.64, *r*_2_ = 0.40*	*r* = 0.07, *r*_2_ = 0.005 ns
100–200 ms	150–250 ms	50 ms	*r* = 0.66, *r*_2_ = 0.43*	*r* = −0.02, *r*_2_ = 0 ns
150–250 ms	150–250 ms	0 ms	*r* = 0.43, *r*_2_ = 0.19 ns	*r* = −0.05, *r*_2_ = 0 ns
200–300 ms	150–250 ms	−50 ms	*r* = 0.29, *r*_2_ = 0.08 ns	*r* = −0.15, *r*_2_ = 0.02 ns
0–100 ms	100–200 ms	100 ms	*r* = 0.08, *r*_2_ = 0 ns0	*r* = 0.047, *r*_2_ = 0 ns
50–150 ms	100–200 ms	50 ms	*r* = 0.11, *r*_2_ = 0.01 ns	*r* = −0.02, *r*_2_ = 0.0 ns
100–200 ms	100–200 ms	0 ms	*r* = 0.10, *r*_2_ = 0.01 ns	*r* = −0.02, *r*_2_ = 0.04 ns
150–250 ms	100–200 ms	−50 ms	*r* = −0.12, *r*_2_ = 0.01 ns	*r* = −0.31, *r*_2_ = 0.10**
200–300 ms	100–200 ms	−100 ms	*r* = −0.17, *r*_2_ = 0.03 ns	*r* = −0.34, *r*_2_ = 0.11**

### Time course of complex spike suppression

Direct stimulation of the N-O pathway results in inhibition of the IO, mediated by release of GABA onto IO synapses. Because the release of GABA is mainly asynchronous (Best and Regehr, 2009), there is an unusually long delay between activation of the N-O pathway and the maximum inhibition of the IO (Hesslow, 1986; Svensson et al., 2006). If, as we have argued, Purkinje cell activity influences complex spike activity via the N-O pathway, then there ought to be a delay between the Purkinje cell CR and the complex spike suppression.

**Figure 5:**
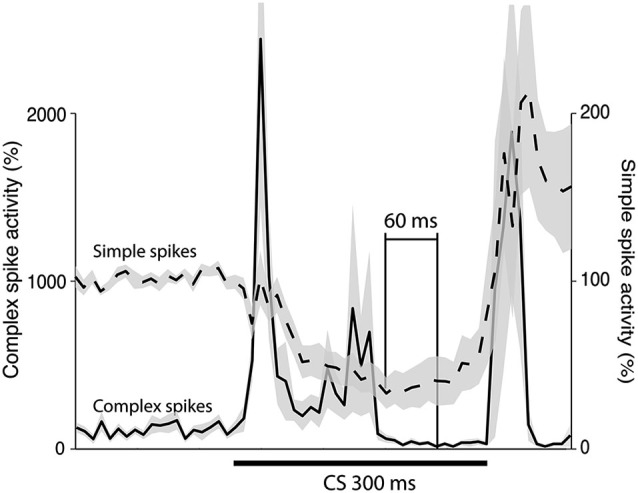
**Simple and complex spike activity after training.** The plot shows the activity (mean ± SEM) of 12 Purkinje cells in the last 100 trials of acquisition (*n* = 8), or in the first 100 trials of extinction (*n* = 4). For each cell the data was binned in 10 ms bins and then averaged over all trials. After this had been done for each cell the average profiles illustrated in the figure were constructed. The minimum was defined as the bin with the lowest firing frequency. For the complex spikes there were several bins with zero activity. The minimum in the figure is the earliest of these points.

To estimate the delay between the Purkinje cell CR, and the suppression of the complex spike frequency we averaged complex spike activity in all cells during the last 100 trials of acquisition and the first 100 trials of extinction into 10 ms bins. In the cells that were recorded during both acquisition and extinction we only looked at acquisition. Following Jirenhed et al. (2007), a suppression was defined as a period in which complex spike activity was below 50% of the background activity, for at least 50 ms. The maximum suppression was defined as the bin, within a suppression, where complex spike activity was at its minimum. Simple spike activity reached its minimum level 180 ms after CS onset. In contrast complex spike activity reached its lowest level 240 ms after CS onset. Moreover, the activity remained at a low level throughout and sometimes beyond the duration of the CS (Figure 5). This 60 ms delay is consistent with the long delay of N-O inhibition and it corroborates the finding that the correlation between simple and complex spike activity was strongest when the simple spike time window preceded the complex spike time window by at least 50 ms (Table 1). At the very least, the results suggest that simple spike activity influences subsequent complex spike activity, rather than the other way around.

### Complex spike activity following training with short ISIs

**Figure 6:**
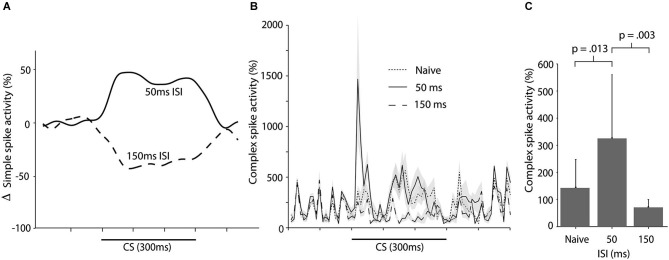
**Simple and complex spike activity following training with short ISIs.** **(A)** Smoothed, mean change in simple spike firing rate as a result of training with either a 50 ms ISI or a 150 ms ISI. Figure adopted from Wetmore et al. (2014; Figure 3D). **(B)** Complex spike activity (mean ± SEM), during the CS in naïve cells (*n* = 15), cells trained with a 50 ms ISI (*n* = 11), and cells trained with a 150 ms ISI (*n* = 10) ISI. For each session the data was binned in 10 ms bins and then averaged over all trials. Activity is expressed as a percentage of the background activity, defined as the activity in the second preceding the CS onset. **(C)** Histogram illustrating the average complex spike frequency and standard deviation (error bars) in naïve cells and in cells trained with either a 50 ms or 150 ms ISI, in the first 100 ms of the CS.

We have recently shown that training with a 50 ms ISI results in increased simple spike activity during the CS (Wetmore et al., 2014; Figure 6A). Here we wanted to examine whether this increase in simple spike activity resulted in a corresponding increase in complex spike activity. To test this, we analyzed 35 CS alone sessions from 20 cells in 16 animals. These CS alone sessions were recorded at regular intervals, interspersed throughout training with different ISIs. There was no statistically significant difference between complex spike activity in naïve cells, and cells trained with a 50 ms ISI, over the whole CS period, *t*_(24)_ = 1.577, *p* = 0.13 (Figure 6B). However, an analysis confined to the first 100 ms of the CS, did reveal a statistically significant increase in complex spikes between naïve cells and cells trained with a 50 ms ISI, *t*_(24)_ = 2.68, *p* = 0.013 (Figure 6C).

In contrast, training with a 150 ms ISI, which supports acquisition of Purkinje cell CRs, lead to reduced complex spike activity when compared with naïve cells (and cells trained with a 50 ms ISI). This suppression was apparent in the first 100 ms of the CS, *t*_(23)_ = 2.11, *p* = 0.046, and over the entire CS period, *t*_(23)_ = 3.57, *p* = 0.002 (Figures 6B,C). Note that, although training with a 50 ms ISI resulted in increased complex spike activity in the first 100 ms of the CS, there was no suppression of complex spikes in the latter part of the CS (Figure 6B). This reaffirms the conclusion that the complex spike suppression seen during training with a 300 ms ISI, is not a rebound inhibition caused by the CS facilitated complex spikes. If this had been the case, then such a rebound should have been present following training with a 50 ms ISI as well.

### Oscillations

**Figure 7:**
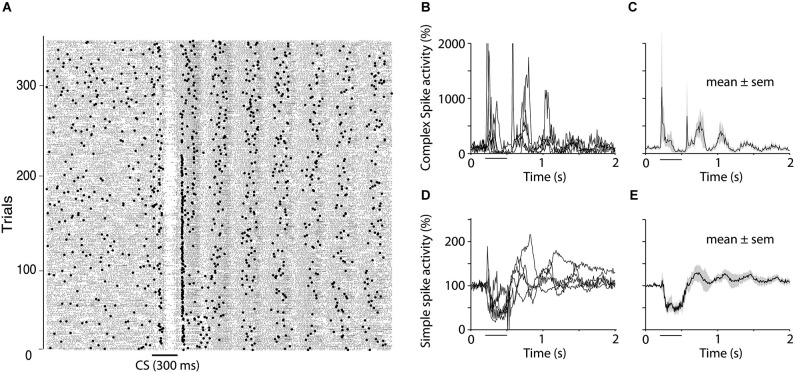
**Oscillations of simple and complex spike activity.** **(A)** Raster plot showing simple spikes (small dots) and complex spikes (larger dots) in one Purkinje cell during extinction (CS alone presentations). The plot shows the rebound in both simple and complex spikes that often occurs following the complex spike suppression as well as the subsequent oscillations that were seen in 50% of the cells recorded. **(B**–**E)** Five cells, which, based on visual inspection of raster diagrams, showed clear oscillations after the CS. Four of these cells were recorded during extinction and one was recoded in the acquisition phase. (**B** and **D**) shows traces from individual cells for complex spikes and simple spikes respectively whereas (**C** and **E**) shows mean ± SEM of these five cells.

In addition to the results presented above, we observed that the suppression of complex spikes at the end of the CS, was often followed by oscillations in both simple and complex spike activity. Complex spikes elicited by the US were not the cause of these because they also occurred on CS alone trials. Figure 7 shows simple and complex spike activity in five cells that exhibited clear oscillations in complex spike activity. The relative timing of the oscillations in simple and complex spikes was inconsistent. The first peak in complex spike activity occurred 60 ms after the first peak in simple spike activity, which is consistent with the delay between the simple and complex spike suppression during the CS. However, subsequent peaks did not follow this pattern. For example, the second peak in complex spike activity came before the second peak in simple spike activity.

## Discussion

Consistent with earlier reports (Sears and Steinmetz, 1991; Hesslow and Ivarsson, 1994), the evidence presented in this paper shows that the IO is suppressed following the emission of a CR. In addition, the evidence shows that this suppression is a consequence of training. This observation, together with the correspondence between the time course of the Purkinje cell CR and the complex spike suppression, supports the view that the suppression of the IO is caused by Purkinje cell CRs disinhibiting the CN and activating the N-O pathway.

To what extent do the firing patterns observed here agree with observations in different setups? While decerebrated ferrets have an intact cerebellum and are capable of acquiring behavioral CRs, if they are not curarized (Hesslow and Ivarsson, 1994), there is a possibility that decerebration can disrupt other brain areas that can affect cerebellar activity. For example, the CN send an excitatory projection to the Nucleus of Darkschewitsch in the midbrain, which in turn sends an excitatory projection to the IO (De Zeeuw and Ruigrok, 1994). If this pathway was damaged, it could alter IO activity, which would affect the results. We have no histological data from these animals, that allow us to examine this possibility, and thus cannot exclude it with certainty. Yet comparison with cats, which have a brainstem very similar to ferrets, suggests that it is unlikely that such damage has occurred in our experiments.

### Variability of complex spike recovery during extinction?

Although extinction is associated with a recovery of simple spike firing during the CS (Jirenhed et al., 2007), changes in complex spike activity were markedly varied in this phase. Only five of the nine cells recorded during extinction had higher activity in the last bin of 50 trials, when compared to the first bin. In the four remaining cells complex spike activity either did not change or increased during extinction. An even more puzzling observation was the negative correlation between simple and complex spike activity in the un-pooled extinction data. Why did the data gathered during extinction deviate from the rest of the dataset?

One possibility is that there really is no difference and that if we had been able to record the cells for longer time periods, we would have seen increased complex spike activity. In support of this view, four of the five cells we recorded for 650 trials, or more, did exhibit increased complex spike firing. Perhaps the biochemical processes responsible for acquisition of the Purkinje cell CR continue for some time after switching to the extinction protocol. Still, this would not explain the absence of a correlation between simple and complex spike activity, which was present during the acquisition phase.

Another possibility is that the variable complex spike recovery during extinction reflects a difference in simple spike activity in other microzones. We know that a cell group in the anterior interpositus nucleus that controls a specific muscle, receives convergent Purkinje cell input from several microzones in the C1, C3 and Y zones (Apps and Garwicz, 2005). This was confirmed in a mapping study of cerebellar cortex of cats, where four microzones controlling blink were found (Hesslow, 1994a), and probably also holds for ferrets (Garwicz, 1997). Our Purkinje cell recordings are all from the same C3 area, but if the extinction process were slower in other microzones, the disinhibition of the nuclear cells and the consequent suppression of the IO would last longer.

Yet another possibility is that acquisition induces plasticity in the CN or the IO, which is not as easily reversed, as the plasticity in the Purkinje cells. The fact that re-acquisition is faster than acquisition, on a behavioral level (Napier et al., 1992), as well as in the Purkinje cells (Jirenhed et al., 2007), shows that extinction is not a mirror image of acquisition (Mauk and Ohyama, 2004). It has been suggested that residual plasticity in the CN is responsible for this rapid re-acquisition or savings (Medina et al., 2001). Could it be that the relative inflexibility of complex spike activity during extinction is a reflection of this residual plasticity?

### Latency between simple spike and complex spike suppression

Direct stimulation of the N-O pathway results in inhibition of the IO. According to previous estimates, this inhibition peaks 30 ms after stimulus onset in ferrets and after ~50 ms in cats, though the effect persists for several 100 ms (Hesslow, 1986; Svensson et al., 2006; Bazzigaluppi et al., 2012; Bengtsson and Hesslow, 2013). We estimated the delay using two different methods. First we plotted the Purkinje cell CR and the complex spike suppression on top of each other (Figure 5). Consistent with the long N-O delay, the minimum complex spike activity occurred 60 ms after the minimum simple spike activity. Furthermore, the complex spike suppression remained strong throughout the CS. Further corroborating evidence was found when we analyzed the correlation between simple and complex spike activity, in different parts of the CS. As shown in Table 1, the correlation was highest when the simple spike window preceded the complex spike window by 50–150 ms. In contrast, complex spike activity did not correlate with subsequent simple spike activity. Our estimate of the N-O delay is longer than the 30 ms suggested by previous results (Svensson et al., 2006), but shorter than the very long delays proposed by Bazzigaluppi et al. (2012). However the 25–40 ms figure is based on direct electrical stimulation of the N-O pathway and it is possible that in the absence of direct N-O stimulation temporal summation at the CN or IO level is required to achieve the same IO suppression, and if so, this could explain the extra delay.

What is the functional significance of this unusually long delay? One plausible suggestion is that it maximizes the inhibitory effect of the CR on the US (Lepora et al., 2010). The Purkinje cell CR following training with a 300 ms ISI, peaks approximately 180 ms into the CS (Jirenhed et al., 2007). The fact that the Purkinje cell CRs peak before the behavioral CRs is consistent with the suggestion that the Purkinje cell CR generates the behavioral CR. Moreover, the long delay in the N-O pathway is consistent with the idea that Purkinje cell CRs inhibit the US. If there had been no delay in the N-O pathway, the inhibition would have occurred too early. In essence, the N-O delay allows for inhibition of the US signal once learning has been achieved (Bengtsson and Hesslow, 2006; Lepora et al., 2010). This hypothesis assumes that, even though the minimum level of complex spike activity occurred 240 ms after the CS onset, there is still a substantial IO suppression after 300 ms. Based on Figure 2F, from which it is clear that the complex spike suppression persists throughout and even beyond the CS, this assumption appears to be justified.

### CS facilitated complex spikes

In the majority of the cells recorded, we observed an increase in complex spike activity during the first 100–150 ms of the CS. Some cells had 10 times higher complex spike activity in the beginning of the CS compared to background, whereas other cells exhibited no change. Why did the CS lead to an increase in complex spikes? One possibility is that the CS directly or indirectly excited the IO. The observation that the CS sometimes induces a 20% increase in simple spike activity (Jirenhed et al., 2007), together with the fact that the cerebellum has a modular organization, suggests another possible explanation for the CS facilitated complex spikes. Increased firing in a large number of Purkinje cells could lead to disinhibition of the IO, via the N-O pathway, which in turn could increase complex activity. However, the fact that some CS facilitated complex spikes appeared after only 10 ms suggests that this cannot be the whole explanation.

Whatever the cause, several observations show that the CS facilitated complex spikes did not influence the development of the complex spike suppression. First, in the two cells where CS facilitated complex spikes were absent, complex spike activity was still suppressed (Figures 3D,E). Second, although CS facilitated complex spikes were present throughout the acquisition and extinction phases, the complex spike suppression developed gradually. Third, the complex spike suppression was present even after we excluded all trials with complex spikes in the early part of the CS (Figure 4).

### Short ISIs

Purkinje cells trained with a 50 ms ISI, instead of a Purkinje cell CR, develop an increase in simple spike activity (Wetmore et al., 2014). Consistent with the hypothesis that simple spike activity influences complex spike activity via the N-O pathway, there was no decrease in complex spike activity following training with a 50 ms ISI. In contrast, a 150 ms ISI, which does support CR acquisition, did lead to a suppression of complex spikes. It thus appears that the complex spike suppression only occurs after training with an ISI that produces a Purkinje cell CR. Because we did not have as much data for the short ISI conditions, we could not estimate the relative timing of the simple and complex spike activity as precisely. Nevertheless, compared to untrained cells and cells trained with a 150 ms ISI, the cells trained with a 50 ms ISI did exhibit increased complex spike firing in the first 100 ms of the CS, suggesting that simple spike activity can modulate complex spike activity in a bidirectional manner.

### Oscillations in simple and complex spike activity

Oscillatory patterns of neuronal activity, have been observed in many different parts of the brain, including the IO (Llinás and Yarom, 1986; Llinás, 1988). In the IO, these membrane potential oscillations are thought to rely on interactions between neurons connected by gap junctions. The properties of these gap junctions, and hence the interactions between neurons in the IO, in turn depend on activation of glutamatergic NMDA receptors (Mathy et al., 2014), as well as GABAergic input from the CN (Lefler et al., 2014). We observed oscillations in simple and complex spike activity in 5 of 12 cells after the CS (Figure 7). The first peak in complex spike activity occurred 60 ms after the first peak in simple spike activity, which is consistent with a N-O mediated disinhibition of the IO. However, the fact that subsequent peaks did not conform to this pattern suggests that other mechanisms are involved. The frequency of the oscillations resembled the 1–10 Hz sub-threshold oscillations previously described in the IO (Lampl and Yarom, 1993; De Zeeuw et al., 2011), yet, the fact that we did not see any oscillations prior to CS onset, in accordance with previous reports (Keating and Thach, 1995, 1997), suggests that sub-threshold oscillations cannot be the sole cause of the observed complex spike oscillations. Another, not necessarily opposing, possibility is that the oscillations are related to behavioral oscillations, which have been observed in finger movements (Vallbo and Wessberg, 1993), as well as eyelid movements (Gruart et al., 2000). Since our animals were paralyzed while recording Purkinje cell activity, we could not determine whether the oscillations in simple and complex spike activity were associated with similar oscillations in the activity of the muscles controlling the eyelid.

### The olive as a movement timer

It has been proposed by some authors that the climbing fiber system functions as a generator of temporal patterns and that these patterns can be used for movement timing (Llinás et al., 2002; Jacobson et al., 2008). Conditioned eyeblinks are prime examples of adaptively timed movements generated by the cerebellum. If the olivary timer hypothesis were true, one would therefore expect the olive to fire in advance of the CR in order to assist in its timing. The fact that the occurrence of an adaptively timed Purkinje cell CR does not seem to depend on the presence of a previous complex spike is a strong argument against the suggestion that the climbing fibers are involved in generating timed movements.

### Implications and limitations

There is an apparent contradiction between the positive correlation between simple spike activity and subsequent complex spike activity that we have shown in this paper and the well-established fact that IO activity, outside the normal range, correlates *negatively* with simple spike activity. Apart from passing on the US signal, the IO also controls the Purkinje cell’s intrinsic spike generator. As a result, high frequency stimulation of the IO or climbing fibers will silence the Purkinje cells, whereas lesioning or blocking the IO results in abnormally high levels of Purkinje cell activity (Colin et al., 1980; Cerminara and Rawson, 2004). We do not know what caused the substantial increase in simple spike activity and the absence of complex spikes, after the US stimulation in the acquisition phase (Figures 2A,B). One possibility however is that the US interferes with the intrinsic spike generator, resulting in an abnormal firing pattern. This would explain why the same pattern is not seen during extinction.

Most work on the role of N-O inhibition in feedback regulation of the IO has dealt with the probability of IO firing, but it now seems clear that the number of impulses (Rasmussen et al., 2013), and in consequence the duration (Yang and Lisberger, 2014) of the climbing fiber signal determines the rate and direction of cerebellar learning (see also Kimpo et al., 2014). This raises the question of whether the N-O feedback signal might modulate the olivary response in a graded manner (Najafi and Medina, 2013; Rasmussen and Hesslow, 2014). While this paper provides strong evidence that learned changes in Purkinje cell activity can affect subsequent IO activity, our recordings did not permit us to assess any effect on the shape of the US elicited complex spikes. The reason is that, in most cells, we used climbing fiber stimulation as a US, thus bypassing the IO and N-O modulation. When we did use a peripheral US, the stimulus artifacts usually masked the complex spikes, which made complex spike identification unreliable. Nevertheless, the IO signal appears to be a crucial variable. For similar reasons, we could not compare our results with the recent report that the N-O pathway synchronizes the IO response and makes it more phasic (Hogri et al., 2014). In the light of this, future research ought to test if Purkinje cell CRs can affect, in a graded manner, the intensity or temporal dynamics of the US induced IO response.

## Conflict of interest statement

The authors declare that the research was conducted in the absence of any commercial or financial relationships that could be construed as a potential conflict of interest.
